# Detection and validation of stay-green QTL in post-rainy sorghum involving widely adapted cultivar, M35-1 and a popular stay-green genotype B35

**DOI:** 10.1186/1471-2164-15-909

**Published:** 2014-10-18

**Authors:** Nagaraja Reddy Rama Reddy, Madhusudhana Ragimasalawada, Murali Mohan Sabbavarapu, Seetharama Nadoor, Jagannatha Vishnu Patil

**Affiliations:** Marker-assisted selection Lab, ICAR-Directorate of Sorghum Research (DSR), Rajendranagar, Hyderabad, 500 030 India; ICAR-Directorate of Medicinal and Aromatic Plants Research (DMAPR), Anand, Gujarat 387 310 India

**Keywords:** Stay-green, Sorghum, Post-flowering drought tolerance, Quantitative trait loci, Marker assisted breeding

## Abstract

**Background:**

Sorghum [*Sorghum bicolor* (L.) Moench] is an important dry-land cereal of the world providing food, fodder, feed and fuel. Stay-green (delayed-leaf senescence) is a key attribute in sorghum determining its adaptation to terminal drought stress. The objective of this study was to validate sorghum stay-green quantitative trait loci (QTL) identified in the past, and to identify new QTL in the genetic background of a post-rainy adapted genotype M35-1.

**Results:**

A genetic linkage map based on 245 F_9_ Recombinant Inbred Lines (RILs) derived from a cross between M35-1 (more senescent) and B35 (less senescent) with 237 markers consisting of 174 genomic, 60 genic and 3 morphological markers was used. The phenotypic data collected for three consecutive post-rainy crop seasons on the RIL population (M35-1 × B35) was used for QTL analysis. Sixty-one QTL were identified for various measures of stay-green trait and each trait was controlled by one to ten QTL. The phenotypic variation explained by each QTL ranged from 3.8 to 18.7%. Co-localization of QTL for more than five traits was observed on two linkage groups i.e. on SBI-09-3 flanked by S18 and Xgap206 markers and, on SBI-03 flanked by XnhsbSFCILP67 and Xtxp31. QTL identified in this study were stable across environments and corresponded to sorghum stay-green and grain yield QTL reported previously. Of the 60 genic SSRs mapped, 14 were closely linked with QTL for ten traits. A genic marker, XnhsbSFCILP67 (Sb03g028240) encoding Indole-3-acetic acid-amido synthetase GH3.5, was co-located with QTL for GLB, GLM, PGLM and GLAM on SBI-03. Genes underlying key enzymes of chlorophyll metabolism were also found in the stay-green QTL regions.

**Conclusions:**

We validated important stay-green QTL reported in the past in sorghum and detected new QTL influencing the stay-green related traits consistently. *Stg2*, *Stg3* and *StgB* were prominent in their expression. Collectively, the QTL/markers identified are likely candidates for subsequent verification for their involvement in stay-green phenotype using NILs and to develop drought tolerant sorghum varieties through marker-assisted breeding for terminal drought tolerance in sorghum.

**Electronic supplementary material:**

The online version of this article (doi:10.1186/1471-2164-15-909) contains supplementary material, which is available to authorized users.

## Background

Sorghum [*Sorghum bicolor* (L.) Moench] is an important dry-land cereal of the world providing food, fodder, feed and fuel [1]. Sorghum carries out C_4_ photosynthesis with a specialized Kranz anatomy for efficient carbon fixation, which makes it a well-adapted cereal crop to environments with high temperature and water limitation [2] and emerged as a model crop species for tropical grass genomics [3]. Globally, sorghum crop is grown on 40 m ha area with grain yield productivity of 1400 kg h^-1^ [4]. Among the sorghum growing countries, India ranks first in area with 7.53 m ha with a productivity of 963 kg ha^-1^ where crop is grown in two contrasting cropping seasons. While rainy season (*kharif*) sorghums are grown on wet soil profile with sufficient monsoon rainfall during the months of June-September, the post-rainy season sorghums are cultivated on the stored soil moisture after the *kharif* rains in a vast area of the Deccan Plateau. Therefore, the growth and development of the sorghum crop during post-rainy season is typically dependent on the available soil moisture, which gets depleted over a period with the progress in crop maturity. While rainy sorghum grain is typically used for non-food uses due to grain-mold disease, the post-rainy sorghum grain is used primarily for human consumption. Post-rainy sorghum grain is highly valued for its pearly white, lustrous, bold and clean grain, 98% of which is used for food [5]. Apart from grain, sorghum stover is an important feed in the livestock sector in India particularly in the dry seasons when other feed resources are in short supply [5]. Thus, post-rainy sorghum plays an important role in ensuring food and fodder security for millions of rural families in the semi-arid tropics. In these areas, since rainfall is low and highly erratic, terminal drought stress is the major yield constraint. Moisture stress during post-flowering stage is the most significant yield reducing factor in the semi-arid tropics [6–11]. The economic benefit of successful mitigation of drought damage by developing drought tolerant sorghum varieties was estimated to be US$ 53 million per year [12]. Under diminishing moisture regimes of post-rainy environment, sorghum crop severely suffers from drought-associated root and stalk rots leading to severe crop lodging, besides loss of stover, grain quality, and productivity [8].

In sorghum, stay-green (delayed-senescence) is a post-flowering drought response [13], and is well characterized by the maintenance of green leaves (upper) and green stems although the plants are under severe moisture stress conditions. The genotypes possessing the stay-green trait maintain more photosynthetically active leaf area as compared to senescent genotypes, and continue to fill their grains normally under stress conditions. Stay-green is also associated with resistance to charcoal rot and stalk lodging, superior fodder quality and higher grain yield [7, 8, 13], increased cytokinin concentration [14] and stem sugars in basal nodes [15]. Moreover, the contribution of the stay-green to stable yield production under post-flowering drought has been documented [7]. The genotype BTx642 (formerly B35) has been identified as a useful source of stay-green [10, 16–18] to unravel its genetic and physiological basis and to develop commercial hybrids [13, 19]. Apart from sorghum, stay-green and its basis has been widely studied in rice [20–23], maize [24–28] durum wheat [29–35], *Festuca pratensis* [36], soybean [37, 38] and sunflower [39, 40]. Loss of chlorophyll is the visible symptom of leaf senescence and the stay-green trait reflects impaired or delayed chlorophyll catabolism [41]. Genes involved in chlorophyll biosynthesis and degradation are cloned [42, 43]. Any impairment in the enzymatic steps responsible for chlorophyll metabolism was associated with the expression of stay-green phenotypes [44–47].

Stay-green is a quantitative trait controlled by nuclear genes [48] and different types of stay-green phenotypes were recognized [9, 21]. Some are cosmetic and are not photosynthetically active (non-functional), whereas others are associated with greater biomass accumulation (functional). High intrinsic chlorophyll concentration has also been associated with improved stay-green in sorghum and reduces post-flowering drought induced senescence [49]. B35 has Type A stay-green characterized by delayed onset of leaf senescence [9, 50].

The progress in genetic improvement of post-rainy sorghum for drought tolerance using traditional plant breeding practices has been slow, and the selection has not been much effective due to complex interaction between genotype and environment. Several component traits are involved in terminal drought tolerance, and each component is genetically controlled by many genes. In addition, the action of these genes is confounded by environment and may involve epistatic interactions. This complicates selection for higher grain yield, and therefore adversely affects gain from selection for an elite genetic stock having drought tolerance and high yield potential [51]. Sorghum breeders have been using stay-green trait for indirect selection of drought tolerance since two decades. For instance, potential use of stay-green QTL contributing to plant water use, transpiration efficiency in different genetic backgrounds was reported [52]. More recently, the co-location of stay-green and nodal root angle QTL in sorghum [53] highlights the probable role of roots in retaining leaves green, and offers an opportunity to use molecular breeding strategies to improve drought tolerance through the manipulation of nodal root angles. However, with limited knowledge of genetics and physiology of this trait, the progress in selection for the stay-green trait using traditional breeding methods is slow. Several factors like on-set and intensity of drought stress, growth stage, heat, inability to evaluate stay-green until plants reach physiological maturity influence efficiency of selection [10].

In view of the above difficulties in unravelling genetic mechanisms controlling stay-green character, QTL approach is appropriate to dissect stay-green at genomic level. Earlier QTL studies for stay-green in sorghum detected several genomic regions associated with its expression [10, 16, 18, 54]. Four major QTL namely, *Stg1* (on SBI-03), *Stg2* (on SBI-03), *Stg3* (on SBI-02), and *Stg4* (on SBI-05) are consistent across genetic and environment backgrounds and accounted 53.5% phenotype variance [18]. *Stg1*, *Stg2* and *Stg3* QTL also co-located with chlorophyll content at physiological maturity [18]. Further, [49] reported that these stay-green QTL reduced drought (post-flowering) induced leaf senescence in the recipient senescent genetic background of RTx7000. Several breeding programs were initiated to incorporate stay-green QTL in advanced breeding lines [50, 55]. More recently, [52] tested the performance of stay-green QTL using a set of introgression lines (ILs) providing an insight into operation of stay-green in combination with other component traits of drought tolerance.

Therefore, stay-green trait is very relevant for the improvement of post-rainy sorghums in India as the crop is grown on residual and receding moisture conditions where occurrence of terminal drought coincides with crop maturity and grain filling stage [56]. Any improvement towards delayed senescence with active photosynthesis during post-flowering stage will not only improve grain filling but also improves fodder quality and charcoal rot resistance. Thus, identification of QTL controlling stay-green in the genetic background of post-rainy (rabi) genotype (M35-1) would validate their effect under post-rainy growing conditions, and also increase our genetic understanding of various components of stay-green trait, clarify the relationships of QTL to candidate genes and provide the basis for MAS. The objectives of the present study were to validate the expression of stay-green QTL reported using B35 in the past, and to identify new QTL if any, for the stay-green trait. This study involved a new mapping population developed from a cross between an important post-rainy sorghum inbred variety M35-1, and the stay-green donor B35. Secondly, we report the co-location of genes/key enzymes involved chlorophyll metabolism with QTL for stay-green and grain yield.

## Methods

### Plant material

The experimental material of the study consists of a F_9_ recombinant inbred line (RIL) population (245 RILs) developed from two sorghum parents, M35-1 and B35. M35-1 is highly popular, tall, single gene dwarf, dual-purpose sorghum variety grown for its bold, lustrous grains, excellent stover [57, 58] and yield stability across sowing dates [59].The other parent B35 is a 3-gene dwarf genotype developed from a germplasm accession from Ethiopian origin IS12555 [60] and is known for its slower senescence [61]. B35 is well characterized for its stay-green phenotype and several researchers [10, 17, 18, 62] have identified a number of stay-green QTL involving B35.

### Field evaluation

The RILs and parents were evaluated during three consecutive post-rainy seasons of 2006 (PR06), 2007 (PR07) and 2008 (PR08) at the research farm of the ICAR-Directorate of Sorghum Research (DSR), Rajendranagar, Hyderabad, India. The material was planted in a completely random block design (CRBD) with three replications. The experimental units were one-row plots; with each row 4-m long, plant-to-plant spacing was 15 cm and, space between rows was 0.75 m. The crop was protected from insect pests such as shoot fly, mites and stem borer following plant protection measures. Mean monthly temperature ranged from 30 to 35°C and there were no rainfall received during the cropping seasons (Additional file 1: Table S4).

### Phenotypic observation

The RILs were characterized for nine traits as a measure of stay-green, besides recording grain yield data. All phenotypic measurements were recorded from five randomly tagged plants from each row in each replication. The nine traits studied include

1. *SPAD meter readings at booting* (apparent leaf chlorophyll content at booting, SPADB), measured at boot leaf stage from five tagged plants in a plot at five places on the second leaf from the top with a SPAD-502 chlorophyll meter (*Konica-Minolta, Co. Ltd,* Tokyo, Japan) and average values were calculated for each plot;
2. *SPAD readings at maturity* (apparent chlorophyll content at maturity, SPADM, measured at maturity, similar to SPADB);
3. *Total number of green leaves at booting* (GLB);
4. *Total number of green leaves at maturity* (GLM);
5. *Per cent green leaves retained at maturity* (PGLM in percentage obtained as ratio between GLM to GLB);
6. *Green leaf area at booting* (GLAB in cm^2^ measured as follows: length and width of all the green leaves from the top to bottom was measured, and the area of each leaves were estimated as leaf length × leaf width × 0.70. [63, 64]. The total green leaf area of each tagged plant was calculated by sum of all the measured leaves);
7. *Green leaf area at maturity* (GLAM measured at maturity similar to GLAB),
8. *Per cent green leaf area retained at maturity* (PGLAM in percentage determined as PGLAM = [GLAM/GLAB] × 100, where, GLAB = Green leaf area at booting (cm^2^) and GLAM = Green leaf area at maturity (cm^2^),
9. *Rate of leaf senescence* (RLS in cm^2^ day^-1^ determined as RLS = [GLAB - GLAM]/number of days taken from flowering and maturity (data not provided) and
10. *Grain yield per panicle* (GY, grain weight per panicle after threshing in g).

### Statistical analysis

The software *SAS 9.2* package (Statistical Analysis Systems Institute Inc., Cary, N.C.) was used for statistical analysis of phenotypic data on stay-green traits and grain yield. Trait variances were partitioned using the random effects ANOVA model y = μ + E + G + G × E + error, where E represents environment, G represents genotype, and G × E represents the genotype by environment interaction. The error term includes the variance between row means for the three replicates of each genotype at each season. We used Proc GLM procedure with replication mean data of each trait in each season for studying the effect of genotype (RILs), environment and genotype × environment interactions for observed variance among RILs by residual maximum likelihood algorithm (REML) as suggested [65]. Broad-sense heritabilities (h^2^) and phenotypic correlations were determined at the level of average performance over three seasons using SAS code [66].

### Linkage map construction

The genetic linkage map of M35-1 × B35 reported [67] was used in this study. The linkage map comprised of 237 markers (174 genomic, 60 genic markers, and three morphological markers spanning a genetic distance of 1235.5 cM) was used for QTL analysis. The details on genotyping of RILs, linkage map construction were described in our previous publication [68].

### QTL analysis

The QTL analysis was performed with trait mean values from individual season (PR06, PR07 and PR08) data, and with across season mean data (AV) for each trait as detailed in our recent publication [67]. The identified QTL were designated with italicized symbol composed of a Q, a trait name, a hyphen, name of institute, the symbol for the chromosome in which the QTL is located, and, in cases where more than one QTL controlling a trait were detected in the same LG, they were numbered serially. For instance, the QTL name *QSpadb-dsr06-1* refers to the SPADB QTL identified at DSR on sorghum SBI-06. QTL were classified as major if the phenotypic variance explained was larger than 10%, and minor when it accounted <10% of phenotypic variance [69]. QTL for different traits were declared to be coincident (co-located) when their positions with highest LOD scores (peak) were located in the same markers intervals. The co-location was “positive” when the additive effects had the same algebraic sign (+or -) and “negative” when they had opposite algebraic signs. QTL stably detected under different environments [70] was referred to as constitutive. In the present study, a QTL was said to be consistent when it was detected in more than one season, with average over seasons, in the multi-environment QTL analysis and across genetic backgrounds in earlier reports at the same locus.

### QTL co-location

The genetic linkage map of the present study has been published recently [67]. A comprehensive analysis of sorghum QTL was reported [71] with the projection of 771 QTL relating to 161 traits from 44 QTL studies onto a sorghum consensus map. All the meta- and unique QTL positions relevant to the 10 traits of the study have been projected onto the physical map using the flanking SSR markers of each QTL to determine co-localization of QTL with previous studies (Additional file 2: Figure S1).

### E-mapping of chlorophyll metabolism related genes

Twenty key genes for enzymes involved in chlorophyll biosynthesis [72]) and degradation [73] were *in-silico* mapped on to the sorghum chromosomes by using their physical positions. The information of remaining genes involved in chlorophyll metabolism were searched in the Plant Metabolic Pathways of Gramene (http://pathway.gramene.org/SORGHUM/class-tree?object=Pathways) and the Sorghum Genome Annotation of Phytozome (http://www.phytozome.net/search.php). These genes were later placed on to the physical map of sorghum.

## Results

Trait mean values of parents M35-1 and B35 and their RIL population for nine stay-green traits and grain yield over three seasons were given in Table 1. The parental lines differed for most of the characters except for GLM. However, wider range of variation for the traits in the RIL population, normal distributions with transgressive segregation suggested polygenic inheritance of the traits (Additional file 3: Figure S2). The estimated broad-sense heritability (h^2^) values for traits were moderate to high and ranged from 0.42 to 0.82. The calculated F values of traits in ANOVA analysis showed the presence of significant differences among the RILs and highly significant environmental effects on traits and genotype × environment interactions (Additional file 4: Table S1).

**Table 1:** **Summary statistics for nine stay-green traits and grain yield studied**

Trait	Parental lines		RILs ^a^				h ^2^
	M35-1	B35	Min	Max	Mean	SEM±	
**SPADB**	52.9	60.9	48.2	62.5	55.2	0.19	0.74
**SPADM**	42.0	49.8	36.5	58.3	45.6	0.25	0.42
**GLB (no.)**	9.2	7.9	6.6	10.4	8.4	0.04	0.82
**GLM (no.)**	4.4	4.5	2.7	8.2	5.2	0.05	0.63
**PGLM (%)**	48.2	56.5	37.2	81.7	61.0	0.55	0.57
**GLAB (cm** ^**2**^ **)**	1915.9	1175.4	850.5	2574.7	1527.1	21.6	0.70
**GLAM (cm** ^**2**^ **)**	894.4	945.8	173.6	1432.3	744.6	16.1	0.71
**PGLAM (%)**	47.5	82.4	15.7	85.5	53.0	0.87	0.63
**RLS (cm** ^**2**^ **day** ^**-1**^ **)**	10.7	1.5	1.2	20.9	9.4	0.30	0.49
**GY (g)**	54.8	31.4	12.5	89.2	42.7	0.20	0.53

*SEM* ± standard error of mean, h^2^ heritability based on average performance over three seasons, ^a^Average over three seasons.

Phenotypic correlations between the traits were estimated based on mean values over three seasons (Table 2). Significant correlation coefficients were observed for most trait combinations. GY was positively correlated with GLB, GLM, PGLM, GLAB, GLAM, and RLS. Highest positive correlation of GY was observed with GLAB followed by GLB. Its correlation with GLM, GLAM and RLS were similar. GY was negatively correlated with SPADB and did not show any correlation with both SPADM and PGLAM.

**Table 2:** **Phenotypic correlation co-efficient among 10 traits studied**

	SPADM	GLB	GLM	PGLM	GLAB	GLAM	PGLAM	RLS	GYPP
SPADB	0.30**	-0.12*	-0.15**	-0.12	-0.27**	-0.10	0.026	-0.07	-0.16**
SPADM		0.23**	0.42**	0.35**	0.21**	0.43**	0.383**	-0.12	0.10
GLB			0.55**	0.08	0.76**	0.56**	0.08	0.36**	0.48**
GLM				0.84**	0.58**	0.71**	0.48**	0.04	0.41**
PGLM					0.21**	0.52**	0.57**	-0.19**	0.18**
GLAB						0.71**	0.06	0.46**	0.56**
GLAM							0.68**	0.004	0.40**
PGLAM								-0.55**	0.003
RLS									0.39**

**Significant at <1%; *Significant at 5%.

### QTL mapping

**Figure 1:**
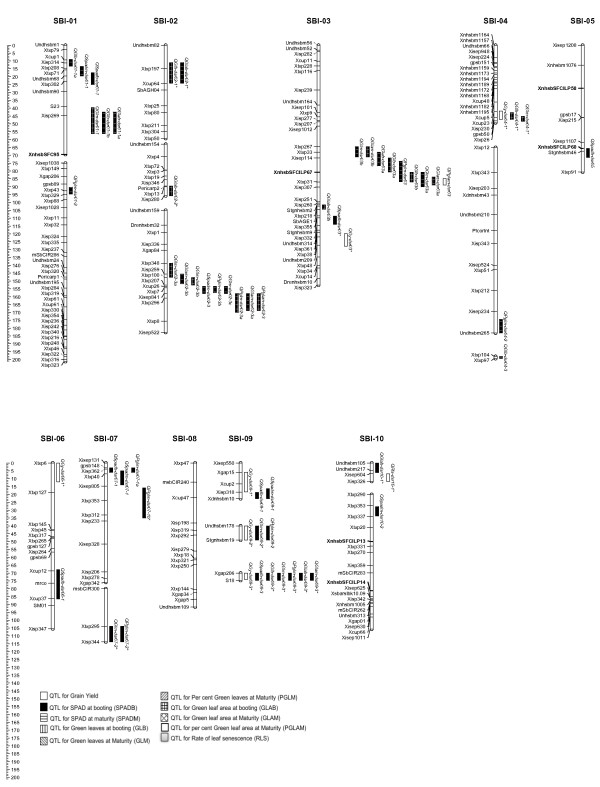
**Genetic linkage map of sorghum showing 61 QTL identified for the grain yield and nine stay-green traits in M35-1 xB35 RIL mapping population.** The useful alleles contributed for the traits by M35-1parent are represented by “asterisk” in the QTL name. The length of the vertical bars indicates 1-LOD support interval. Maximum LOD for each QTL is indicated as a beak on the bar which shows QTL position.

QTL results for 10 traits relating to stay-green and grain yield in the population are shown (Figure 1) and the QTL statistics are summarized in Table 3. QTL for each trait were identified initially by interval mapping, followed by composite interval mapping with co-factors. A total of 61 QTL were detected of which, 47 QTL were detected with LOD threshold of ≥ 3.0, and the remaining 14 QTL (suggestive QTL) were detected with 2.5 to 3.0 LOD.

**Table 3:** **Quantitative trait loci (QTLs) detected for nine stay-green related traits and grain yield studied in M35-1 × B35 RIL population**

Traits	QTL name ^@^	Environment	QTL × E	LG	Position	Left marker	Right marker	LOD	Increased effect	R ^2^(%) ^b^	Additive effect ^a^	Reference
SPAD values at Booting (SPADB)	*QSpadb-dsr09-1*	AV, I, II, III		SBI-09-1	20.7	Xisp318*	Xdhsbm10	6.5	B35	15.0	-1.00	New
	***QSpadb-dsr09-3***	M, AV, III	No	SBI-09-3	2.0	S18*	Xgap206	3.8	B35	9.3	-1.09	[17, 54]
	*QSpadb-dsr05*	AV, I, II		SBI-05	67.4	Stgnhsbm46*	Xtxp091	4.1	B35	8.0	-1.49	[10, 17, 18, 74, 75]
	*QSpadb-dsr06-1*	AV, II		SBI-06-1	76.5	Xcup12*	Xcup37	4.1	B35	6.9	-0.89	New
	***QSpadb-dsr07-1***	M, II	Yes	SBI-07-1	6.0	Xisp362*	Xtxp40	4.8	B35	6.9	-0.83	[18, 76]
	*QSpadb-dsr03*	AV, I		SBI-03	112.0	Xisp355*	Xtxp38	3.2	M35-1	5.0	0.73	New
	*QSpadb-dsr01-1*	AV		SBI-01-1	20.5	Unnhsbm68	Xtxp302*	2.7^#^	B35	3.9	-0.63	[17, 54, 76]
SPAD values at maturity (SPADM)	***QSpadm-dsr10-2***	M, AV, I, II, II	No	SBI-10-2	9.7	Xtxp337*	Xtxp20	6.0	B35	14.1	-1.24	[76]
	***QSpadm-dsr01-1***	M, AV, I	Yes	SBI-01-1	16.5	Xtxp71	Unnhsbm68*	4.2	B35	8.5	-2.18	[76]
	*QSpadm-dsr07-1*	II		SBI-07-1	9.9	Xtxp40*	Xisep0805	4.4	B35	7.8	-0.98	[18, 76]
	*QSpadm-dsr02-3*	AV		SBI-02-3	50.4	Xtxp7*	Xisep0841	3.2	B35	5.3	-0.93	[10, 18, 54, 76]
	*QSpadm-dsr09-1*	II		SBI-09-1	18.7	Xisp318*	Xdhsbm10	2.5^#^	B35	4.2	-0.69	New
Green leaves at booting (GLB)	*QGlb-dsr03a*	AV, I, II, II		SBI-03	75.7	Xisep0114*	XnhsbSFCILP67	6.6	B35	18.7	-0.33	[10, 17, 18, 74–76]
	***QGlb-dsr09-3***	M, AV, II, III	Yes	SBI-09-3	3.0	S18*	Xgap206	5.4	M35-1	18.4	0.29	[17, 54]
	***QGlb-dsr01-1a***	M, AV, I, II	No	SBI-01-1	12.0	Xisp314	Xtxp208*	6.5	B35	15.7	-0.25	New
	*QGlb-dsr01-1b*	III		SBI-01-1	51.5	Xisp269*	XnhsbSFCILP95	2.8^#^	B35	6.6	-0.22	[17, 54, 76]
	*QGlb-dsr04-1*	AV, I		SBI-04-1	46.2	Xisp230*	gpsb050	3.2	M35-1	5.6	0.28	New
	***QGlb-dsr03b***	M, II	Yes	SBI-03	84.8	XnhsbSFCILP67	Xtxp31*	2.7^#^	B35	4.9	**-0.23**	[10, 17, 18, 74–76]
	*QGlb-dsr02-1*	II		SBI-02-1	20.1	Xtxp197*	Xcup64	2.7^#^	M35-1	4.8	0.20	New
	*QGlb-dsr04-3*	II		SBI-04-3	1.0	Xtxp097*	Xtxp104	2.6^#^	B35	3.8	-0.17	New
Green leaves at maturity (GLM)	*QGlm-dsr03a*	AV, I, II, II		SBI-03	84.8	XnhsbSFCILP67*	Xtxp31	8.1	B35	15.9	-0.39	[10, 17, 18, 74–76]
	*QGlm-dsr02-3a*	AV, I, II		SBI-02-3	38.6	Xtxp348	Xisp259*	7.7	B35	13.9	-0.32	[10, 18, 54, 76]
	***QGlm-dsr09-3***	M, AV, I, II	No	SBI-09-3	0.0	S18*	Xgap206	6.4	M35-1	8.7	0.31	[17, 54]
	*QGlm-dsr02-3b*	AV, I		SBI-02-3	45.7	Xtxp100	Xtxp207*	4.2	B35	8.2	-0.38	[10, 18, 54, 76]
	***QGlm-dsr03b***	M, AV	No	SBI-03	67.5	Xtxp267	Xtxp33*	4.7	B35	6.7	-0.23	[54]
	*QGlm-dsr07-2*	AV		SBI-07-2	33.8	Xtxp295	Xisp344*	4.0	M35-1	6.6	0.25	[76]
	*QGlm-dsr01-1*	II		SBI-01-1	47.5	Xisp269	XnhsbSFCILP95*	3.5	B35	5.2	-0.25	[17, 54, 76]
	***QGlm-dsr02-3c***	M, II	No	SBI-02-3	49.4	Xtxp7*	Xisep0841	3.9	B35	5.1	-0.23	[10, 18, 54, 76]
	***QGlm-dsr04-1***	M, II	Yes	SBI-04-1	47.2	Xisp230	gpsb050*	3.7	M35-1	5.0	**0.24**	New
	*QGlm-dsr09-2*	AV		SBI-09-2	6.0	Unnhsbm178	Stgnhsbm19*	2.6^#^	M35-1	4.1	0.21	New
Per cent green leaves retained at maturity (PGLM)	***QPglm-dsr02-3a***	M, AV, I, II, III	No	SBI-02-3	57.9	Xtxp296*	Xtxp8	6.8	B35	14.9	-3.25	[10, 18, 54, 76]
	*QPglm-dsr07-2*	AV, I		SBI-07-2	31.9	Xtxp295	Xisp344*	4.2	M35-1	10.3	2.71	[76]
	*QPglm-dsr07-1a*	AV, III		SBI-07-1	4.0	Xisp362*	Xtxp40	3.5	B35	10.0	-2.84	[18, 74, 76]
	*QPglm-dsr09-3*	AV, I, II		SBI-09-3	3.0	S18	Xgap206*	3.8	M35-1	7.2	4.05	[17, 54]
	*QPglm-dsr01-2*	II		SBI-01-2	17.6	Xtxp43*	Xtxp329	4.6	B35	7.1	-2.15	[17, 54, 76]
	*QPglm-dsr04-2*	I		SBI-04-2	118.1	Xisep0234	Unnhsbm265*	3.6	B35	6.4	-3.82	New
	*QPglm-dsr02-3b*	I		SBI-02-3	49.4	Xtxp7*	Xisep0841	3.1	B35	5.7	-3.65	[10, 18, 54, 76]
	*QPglm-dsr09-2*	AV		SBI-09-2	9.0	Unnhsbm178	Stgnhsbm19*	2.8^#^	M35-1	5.3	2.10	New
	*QPglm-dsr03*	II		SBI-03	83.8	XnhsbSFCILP67*	Xtxp31	2.9^#^	B35	4.9	-1.84	[10, 17, 18, 74–76]
	***QPglm-dsr07-1b***	M, II	Yes	SBI-07-1	25.8	Xisep0805	Xtxp312*	2.8^#^	M35-1	4.9	**1.85**	[18, 74, 76]
Green leaf area at booting (GLAB)	*QGlab-dsr03a*	AV, I, II, II		SBI-03	70.0	Xtxp33*	Xisep0114	6.2	B35	15.5	-125.63	[10, 17, 18, 74–76]
	***QGlab-dsr09-3***	M, AV, II, III	No	SBI-09-3	3.0	S18*	Xgap206	5.2	M35-1	10.6	100.77	[17, 54]
	***QGlab-dsr01-1a***	M, AV, I, II	No	SBI-01-1	50.5	Xisp269	XnhsbSFCILP95*	4.6	B35	9.3	-114.12	[17, 54, 76]
	*QGlab-dsr03b*	I		SBI-03	102.3	Xtxp218*	SbAGE1	2.9^#^	B35	4.9	-92.64	[10, 18, 76]
	***QGlab-dsr10-1***	M, I	Yes	SBI-10-1	3.0	Unnhsbm105*	Unnhsbm217	2.9^#^	M35-1	4.9	98.29	New
	*QGlab-dsr02-1*	II		SBI-02-1	16.1	Xtxp197*	Xcup64	3.2	M35-1	4.8	91.52	New
	*QGlab-dsr02-2*	II		SBI-02-2	27.0	Xtxp013*	Xisp280	3.0	M35-1	4.7	92.81	New
Green leaf area at maturity (GLAM)	***QGlam-dsr03a***	M, AV, I, II, II	Yes	SBI-03	86.8	XnhsbSFCILP67*	Xtxp31	4.7	B35	12.6	**-105.69**	[10, 17, 18, 74–76]
	***QGlam-dsr02-3a***	M, AV, II, III	Yes	SBI-02-3	55.9	Xisep0841	Xtxp296*	3.0	B35	10.7	-61.58	[10, 18, 54, 76]
	***QGlam-dsr09-3***	M, AV, I, II	No	SBI-09-3	0.0	S18*	Xgap206	3.7	M35-1	6.3	89.54	[17, 54]
	*QGlam-dsr03b*	AV, I		SBI-03	68.0	Xtxp267	Xtxp33*	3.5	B35	5.9	-60.56	[10, 17, 18, 74–76]
	*QGlam-dsr02-3b*	AV		SBI-02-3	42.7	Xisp259	Xtxp100*	3.3	B35	5.7	-60.31	[10, 18, 54, 76]
Per cent green leaf area at maturity (PGLAM)	***QPglam-dsr02-3***	M, AV, I, II, II	Yes	SBI-02-3	54.9	Xisep0841	Xtxp296*	5.1	B35	13.1	-5.04	[10, 18, 54, 76]
	***QPglam-dsr03***	M, AV, I, II, II	Yes	SBI-03	87.0	XnhsbSFCILP67*	Xtxp31	3.3	B35	8.4	**-4.30**	[10, 17, 18, 74–76]
Rate of leaf senescence (RLS)	*QRls-dsr10-1*	AV, I, II, II		SBI-10-1	10.9	Xisep0604	Xisp326*	3.3	M35-1	10.5	1.20	New
Grain yield per panicle (GY)	*QGY-dsr06-1*	M, AV, I, II	Yes	SBI-06-1	6.0	Xtxp6*	Xtxp127	6.0	M35-1	11.4	2.68	[75, 77]
	*QGY-dsr09-2*	M, AV, II	No	SBI-09-2	3.0	Unnhsbm178*	Stgnhsbm19	4.2	M35-1	7.3	2.08	[78]
	*QGY-dsr04-1*	AV, I		SBI-04-1	46.2	Xcup23	Xisp230*	3.5	M35-1	6.4	3.37	[77]
	*QGY-dsr09-1*	M, AV, II	Yes	SBI-09-1	11.9	Xgap15	Xcup2*	3.8	M35-1	5.9	**2.29**	[78]
	*QGY-dsr03*	AV, II		SBI-03	124.9	Xisp332	Undhsbm314*	2.5^#^	B35	4.0	-1.92	[77, 79]
	*QGY-dsr09-3*	II		SBI-09-3	0.0	S18*	Xgap206	2.8^#^	M35-1	4.0	1.95	[77, 78]

*Nearest marker to QTL position. Seasons I, II, III, AV (average) and M (multi-season) indicate QTL detected in the seasons Post-rainy 2006, Post-rainy 2007, Post-rainy 2008, across all seasons and in multi-season analysis, respectively; ^a^Additive effect of M35-1. A positive value implies that the M35-1 allele increased phenotypic value, whereas a negative value implies that the M35-1 allele decreased phenotypic value. A QTL effect with bold and underlined indicates inversion of effect in Multi-season QTL analysis; ^b^ R^2^ (%) is percentage of phenotypic variation explained by individual QTL; ^#^Suggestive QTL (LOD <3.0); ^@^QTL in bold are identified in multi-environment QTL analysis also.

### SPAD at booting (SPADB)

Seven QTL for SPADB were identified in the population. Two QTL on linkage groups SBI-09, and a QTL each on SBI-01, SBI-03, SBI-05, SBI-06 and SBI-07 were found. Of the seven, five QTL were identified for mean data over the three seasons and two of them were detected in multi-environment QTL analysis. Increased SPADB values were contributed by the stay-green parent B35 at six QTL. A major QTL (*QSPADB-dsr09-1*) explaining 15% of the phenotypic variance was identified on SBI-09 between the markers Xisp318 and Xdhsbm10. Parent M35-1 contributed for the increased SPADB value at a QTL on SBI-03 between Xisp355-Xtxp38 markers. The phenotypic variance explained by each SPADB QTL ranged from 3.9 to 15%.

### SPAD at maturity (SPADM)

Five QTL were identified for SPADM in the population, and were distributed across five linkage groups with a QTL each on SBI-01, SBI-02, SBI-07, SBI-09, and SBI-10. Of the five, two QTL were identified for mean data over the three seasons and two of them were detected in multi-environment QTL analysis. A major QTL for this trait, *QSpadm-dsr10-2* was identified between SSR markers, Xtxp337 and Xtxp20 on SBI-10 with a LOD of 6.0 explaining 14.1% of phenotypic variance. At all QTL, stay-green parent B35 contributed alleles for increased SPAD value at maturity. The phenotypic variance explained by each QTL ranged from 4.2 to 14.1%. The QTL (*QSpadm-dsr09-1*) on SBI-09 was identified for SPADM co-located with QTL for SPADB.

### Green leaves at booting (GLB)

A total of eight QTL were detected for GLB, and were localized on five chromosomes with two QTL each on SBI-01, SBI-03 and SBI-04 and a QTL each on SBI-02 and SBI-09. Of the eight QTL, four QTL were identified for average performance over the three seasons and three of them were detected in multi-environment QTL analysis. Three major QTL, *QGlb-dsr03, QGlb-dsr09-3*, *QGlb-dsr01-1a* explaining 18.7%, 18.4 and 15.7% of phenotypic variance respectively were identified. The positive alleles for the QTL on SBI-02 near Xtxp197, on SBI-04 near Xisp230 and on SBI-09 near S18, for the increased GLB were contributed by senescent parent M35-1, and at QTL on other linkage groups, M35-1 alleles decreased the trait value. The LOD scores ranged from 2.6 to 6.6 and phenotypic variance ranged from 3.8 to 18.7%.

### Green leaves at maturity (GLM)

A total of ten QTL for GLM were detected in the present population and were distributed onto six linkage groups with three QTL on SBI-02, two on SBI-09 and SBI-03, and a QTL each on linkage groups SBI-01, SBI-04 and SBI-07. Of the ten QTL, four QTL were identified for average performance over the three seasons and four QTL were detected in multi-environment QTL analysis. Two major QTL, *QGlm-dsr03a* on SBI-03, and *QGlm-dsr02-3a* on SBI-02 explaining 15.9% and 13.9% phenotypic variation respectively were identified in the present study. The positive alleles for increased GLM were contributed by senescent parent M35-1 at QTL on SBI-09, SBI-07 and SBI-04 chromosomes and, for remaining QTL on other linkage groups, stay-green parent B35 contributed for increased number of green leaves at maturity. From among 10 QTL for GLM, three QTL were also common to GLB.

### Percent green leaves at maturity (PGLM)

PGLM is an important measure of stay-green and ten QTL distributing on six linkage groups with three on SBI-07, two on SBI-02 and SBI-09 linkage groups, and a QTL each on SBI-01, SBI-04 and SBI-03 were identified. Of the ten, four QTL were identified for average performance over the three seasons and two QTL were detected in multi-environment QTL analysis. Three major QTL, *QPglm-dsr02-3a* (14.9%), *QPglm-dsr07-2* (10.3%) and *QPglm-dsr07-1a* (10.0%) explained larger phenotypic variance. The positive alleles for increased PGLM were contributed by senescent parent M35-1 at QTL on SBI-07 (near Xisp344), SBI-09 and a QTL on SBI-07 (near Xtxp312) chromosomes, and alleles for the QTL on other chromosomes were contributed by stay-green parent B35. Phenotypic variance explained by each QTL ranged from 4.9 to 14.9% and the LOD scores ranged from 2.8 to 6.8.

### Green leaf area at booting (GLAB)

A total of seven QTL, two QTL each on SBI-02 and SBI-03, and a QTL each on SBI-01, SBI-09 and SBI-10 were located for GLAB. Two major QTL, *QGlab-dsr03a* explaining 15.5% phenotypic variation and, *QGlab-dsr09-3* explaining 10.6% phenotypic variation were identified in the population. Of the seven, five QTL were identified for mean data over seasons and two of them were detected in multi-environment QTL analysis. Out of seven QTL that were identified for this trait, three QTL were identified for average performance over the three seasons and three QTL were detected in multi-environment QTL analysis. The alleles for increased GLAB were contributed by stay-green parent B35 at three QTL regions. Interestingly, at QTL positions, *QGlab-dsr09-3, QGlab-dsr010-1*, *QGlab-dsr02-1*, and *QGlab-dsr02-*2, the alleles for increased GLAB were contributed by senescent parent M35-1 also. The phenotypic variance explained by each QTL ranged from 4.7 to 15.5% and the LOD scores ranged from 3.0 to 6.2.

### Green leaf area at maturity (GLAM)

Five QTL were detected for GLAM in the population with two QTL each on SBI-02 and SBI-03, and a QTL on SBI-09. Four QTL were identified for mean performance over the three seasons and three QTL were detected in multi-environment QTL analysis. The alleles from B35 associated with higher green leaf area at maturity were at the QTL regions *QGlam-dsr03a* and *QGlam-dsr03b* on SBI-03, *QGlam-dsr02-3a* and *QGlam-dsr02-3b* on SBI-02, while the alleles for this parent associated with less GLAM at the QTL, *QGlam-dsr09-3.* The phenotypic variation explained ranged from 5.7 to 12.6%. A QTL, *QGlam-dsr03a* was detected on SBI-03 and explained 12.6% of phenotypic variance.

### Percent green leaf area at maturity (PGLAM)

Two QTL each on SBI-02 and SBI-03 for PGLAM in the population were detected. A major QTL, *QPglam-dsr02-3* was detected on SBI-02 explaining 13.1% of phenotypic variance. Both the QTL were identified for average performance over the three seasons as well detected in multi-environment QTL analysis. The positive alleles were derived from B35 parent in the case of both QTL. The QTL were detected with a LOD of 3.3 and 5.1 explaining 8.4 and 13.1% phenotypic variance respectively.

### Rate of leaf senescence (RLS)

A single QTL was identified for RLS on SBI-10 explaining 10.5% of phenotypic variance, and the alleles at this QTL region were derived from senescent parent M35-1 which caused increased rate of leaf senescence (RLS).

### Grain yield (GY)

For GY, a total of six QTL were detected with a distribution of three QTL on SBI-09 and a QTL each on SBI-03, SBI-04 and SBI-06 were detected. Out of six QTL, five QTL were detected for average performance over three seasons and three of them were detected in multi-environment QTL analysis. These QTL individually explained 4.0 to 11.4% of phenotypic variance, and together accounted for 39.0% of the grain yield variation in the population. At majority of the QTL positions, the positive alleles were derived from high yielding parent M35-1. However, a QTL on SBI-03 for grain yield, the positive allele for increased grain yield was contributed by low yielding parent B35. A major QTL, *QGy-dsr06-1* was detected on SBI-06 which explains 11.4% of phenotypic variance with a LOD of 6.0. Of six QTL, detected for grain yield, map position of five QTL coincided with map position of QTL for PW. The increasing effects of QTL alleles influencing the trait at these common QTL regions were also from M35-1.

## Discussion

Development of cultivars tolerant to terminal drought is one of the important goals of sorghum breeding worldwide, and is especially true for the post-rainy sorghums in India. Broadening genetic diversity by employing diverse alleles for post-rainy adaptation drought tolerant traits like stay-green is required. Identification of genetic factors involved in stay-green would establish a base for genetic improvement for drought tolerance. Earlier studies have identified important stay-green QTL. The present study focused on mapping and validating stay-green QTL using considerably a large (247) RIL mapping population derived from M35-1 x B35 cross. The post-rainy season is ideal for evaluating the expression of stay-green trait as the crop depends entirely on stored soil moisture, and undergoes a long, progressive stress with high demands for evaporation [80] during grain filling. Responses of plant to drought stress are undoubtedly affected by time of occurrence and intensity of stress [81], which makes the genetic analysis of drought resistance traits more complicated. Therefore, the pooled average of three seasons data along with individual seasons data were used for QTL analysis. The present study resulted in the identification of 61 QTL for nine traits related to stay-green measure and grain yield.

The significant phenotypic values for various stay-green related traits confirmed the inherent potential of the stay-green parent B35 [17, 48, 61]. High heritability (h^2^) values observed for the component traits of stay-green and grain yield in the present study substantiated high h^2^ observed for stay-green trait [10, 82, 83] and for grain yield [64].

GLB and GLAB are positively correlated. Both individually showed highest positive correlation with GY indicating their greater contribution towards filling the sink under terminal drought. GLB also positively correlated with GLAM which in turn showed positive correlation with GY and SPADM. Therefore, GLB that is easy to measure could act as criteria of selection for higher grain yield under terminal drought. GY and PGLAM were not correlated though GY showed significant association with GLAB and GLAM. This could be due to GLAB, which showed no association with PGLAM. Presence of more green leaves or greater green leaf area either at boot or at maturity contributed for higher photosynthesis and better availability of food reserves for higher grain filling and enhanced grain yield. RLS is positively associated with GY, GLB, GLAB and negatively correlated with PGLM and PGLAM. Positive correlation between RLS and GY indicate physiological processes related to grain filling during leaf senescence as the plants undergo drought stress. Higher leaf senescence indicated higher translocation of food reserve from leaves for better grain filling and increased grain yields.

SPADB, a measure of chlorophyll content at booting was negatively correlated with GY. This could be due to its negative association with GLB, GLM and GLAB, which were positively associated with GY. Thus, selecting for higher chlorophyll content at booting may not be useful. However, one may select for SPADM (chlorophyll at maturity) as it showed significant association with GLB, GLM, PGLM, GLAB, GLAM and PGLAM as most these traits individually correlated significantly with GY.

Of the 61 QTL identified for nine stay-green traits and grain yield, 35 QTL (57.3%) were significantly identified in combined analysis and at one or more locations and averages across three seasons, while 25 QTL (45.4%) were detected with only one of four datasets indicating consistency of QTL detection. However, six of the ten QTL for PGLM and six of the ten QTL for GLM were identified in single environment. These QTL may be environment specific since stay-green expression at maturity is triggered by the quantity or intensity of drought stress at physiological maturity. Nevertheless, some of the inconsistent QTL identified for PGLM and GLM were co-located with stay-green QTL that were consistently identified in more than one location from previous studies and, or co-located with QTL for other component traits of stay-green such as SPADB, SPADM, GLB, GLM, GLAB and GLAM which were identified in more than one season, indicating that these QTL can also be considered consistent since these were highly related.

Earlier QTL studies using B35 as stay-green donor [10, 16–18, 54] identified four major QTL viz., *Stg1*, *Stg2*, *Stg3*, and *Stg4* and were ranked in the order *Stg2* > *Stg1* > *Stg3* > *Stg4* based on their phenotypic contribution [10, 49, 52]. *Stg1* and *Stg2* located on SBI-03 explained 20% and 30% [10, 62] while *Stg3* on SBI-02 accounted for 16% and *Stg4* on SBI-05 controlled 10% of the phenotypic variance [62].

Introgression lines with *Stg2* displayed lower rate of senescence consistently over locations and seasons [49]. A meta-QTL was projected to co-locate with *Stg2* QTL [71]. In the present study too, nine QTL were observed within the confidence interval of *stg2* QTL for six measures of stay-green. Across measured traits, this QTL accounted for an average of 10% trait variance indicating its major role in delayed leaf senescence. This QTL functionally maintained more green leaf area (stay-green) by slower rate of leaf senescence [49]. It is important to note that stay-green parent B35 contributed favourable allele for increased trait values at each of these QTL. Across seasons, B35 showed significantly lesser (x10) rate of leaf senescence compared to senescence parent, M35-1. Thus as reported earlier, *Stg2* is an important QTL for maintaining higher green leaf area contributing for slow senescence. This QTL was also reported to contribute for higher green leaf area at anthesis and maturity by [64] and for percent green leaf area at 45 days after flowering by [76] in different genetic backgrounds. This demonstrates that the expression of *Stg2* QTL was consistent, and forms an important QTL for marker-assisted improvement of post-rainy sorghum lines for terminal drought tolerance.

Similarly, *Stg3* was also identified as a meta QTL [71]. Several workers [10, 18, 54, 76] reported this QTL using different donor parents. [76] detected this QTL as a measure of per cent green leaf area at 15, 30 and 45 days after flowering. Since the QTL was detected in different genetic backgrounds and locations, this QTL with stable expression also important for improving terminal drought tolerance. In the present study, QTL for five stay-green traits viz., SPADM (*QSpadm-dsr02-3*), GLM (*QGlm-dsr02-3a* and *QGlm-dsr02-3a*), PGLM (*QPglm-dsr02-3a and QPglm-dsr02-3b*), GLAM (*QGlam-dsr02-3a and QGlam-dsr02-3b*) and PGLAM (*QPglam-dsr02-3*) were found to co-locate with *Stg3* QTL. QTL for chlorophyll content [10] and a major QTL for root dry weight [71] were co-located with *Stg3. Stg3* could be involved in increased root mass for better moisture capture. As the pattern of water use after anthesis affects grain yield [84], *Stg3* QTL may positively influence grain yield under post-anthesis drought stress.

*QPglm-dsr02-3a*, a major QTL explaining 15% was identified on SBI-02 with the key stay-green QTL *StgB* reported earlier. *StgB* introgression lines in the background of R16 (another post-rainy sorghum line) showed improved stay-green trait [50], and modified the proportion of water extracted before and after anthesis in the S35 background [52]. *StgB* increased total biomass (grain + stover) across genetic backgrounds of S35 and R16. Interestingly *StgB* operated through different physiological mechanisms to maintain stay-green depending on the genetic background. In S35, *StgB* increased water extraction while in R16 background it increased transpiration efficiency. Therefore, stay-green was possibly related to different components such as transpiration efficiency, water extraction capacity or both, both of which are likely to be an aggregate of different mechanisms [52]. R16 is the pedigree derivative of M35-1, the parent of the present population. It is therefore possible that in the present study also *StgB* contributed for stay-green through increased transpiration efficiency. Hence, *StgB* may be one of the key stay-green QTL for MAS to improve post-flowering drought tolerance.

*Stg1* and *Stg4* reported by [10] and [18], were also projected as meta QTL [71]. Several workers reported QTL coinciding with *Stg1* [17, 64, 76, 82]. In this study, one QTL each at *Stg1* interval (*QGlab-dsr03b* ) and *Stg4* interval (*QSpadb-dsr05*) were co-located. [83] also identified QTL *St5* corresponding to *Stg4*.

Four meta QTL for stay-green viz., *QSTG_meta1.1*, *QSTG_meta2.1*, *QSTG_meta3.1* and *QSTG_meta4.1* were projected on SBI-01 [71]. In this study, six stay-green QTL (*QSpadb-dsr01-1*, *QSpadm-dsr01-1*, *QGlb-dsr01-1b*, *QGlm-dsr01-1b*, *QPglm-dsr01-2* and *QGlab-dsr01-1a*) were co-located overlapping with meta QTL *QSTG_meta2.1*. QTL overlapping with *QSTG_meta2.1* have been identified [17, 76]. Thus, this QTL was consistent in its expression across genetic backgrounds and locations. B35 contributed positive alleles for stay-green at this meta QTL [17] which was validated in the present study also. Similarly, [76] also reported the contribution of favourable alleles from E36-1, another well-known source of stay-green in sorghum at this meta QTL. It is therefore likely that the allele at this stay-green QTL is different in stay-green donors from the senescent sorghum genotypes. SBI-01 also hosted a major QTL *QGlb-dsr01-1a* with 15% trait expression, which was not reported earlier.

A major QTL cluster of eight QTL was observed within two Mb on SBI-09. Interestingly, parent M35-1 contributed positive alleles for seven of the traits. Across stay-green traits, this QTL accounted for 9% of trait phenotypic expression. This QTL was also reported [17, 54, 83]. Similar to our study, [54] also reported the contribution of favourable allele from senescent parent, QL41 at this QTL. Due to its consistent expression in different genetic backgrounds and in diverse test locations, this QTL is also a key QTL member of stay-green. A meta QTL has been projected to co-locate at this genomic position [71].

Two meta QTL (*QSTG_meta1.7* and *QSTG_meta2.7*) have been projected on SBI-07. In the present study, the QTL for SPADB (*QSpadb-dsr07-1*), SPADM (*QSpadb-dsr07-1*) and PGLAM (*QPglam-dsr07-*1a and *QPglam-dsr07-*1b) were found co-located with the *QSTG_meta1.7*, while the QTL for GLM (*QGlm-dsr07-2*) and PGLM (*QPglm-dsr07-2*) were co-located with *QSTG_meta2.7*. Notably, M35-1 parent contributed favourable allele for increased trait values of QTL in this clusters. Similarly, another meta QTL (*QSTG_meta1.10*) has been projected on SBI-10. In the present study, the QTL for SPADM (*QSpadm-dsr10-2*) was co-located with this meta QTL. These QTL could be considered as reliable as they were identified as meta-QTL, and form valuable genetic loci for stay-green in sorghum.

### Co-location QTL for stay-green and grain yield

Of the six QTL identified for grain yield, four were co-located with stay-green QTL. Co-localization of stay green and grain yield QTL under drought stress, suggesting that the gene (s) underlying stay green may also result in enhanced yield performance under drought stress [16]. *QGY-dsr09-2* co-located with QTL for GLM (*QGlm-dsr09-2*) and PGLM (*QPglm-dsr09-2*). *QGY-dsr09-2* QTL was reported to be meta-QTL [71], and was co-located with QTL for grain yield component traits such as panicle weight (*QPW-dsr09-2*), test weight (*QTW-dsr09-2*), panicle length (*QPl-dsr09-2*) and plant height (*QPh-dsr09-2*) ([67]). *QGy-dsr09-1* QTL on SBI-09 was co-located with major QTL for SPADM (*QSpadm-dsr09-1*) whereas *QGy-dsr09-3* QTL was co-located with QTL for SPADB (*QSpadb-dsr09-3*), GLB (*QGlb-dsr09-3*), GLM (*QGlm-dsr09-3*), PGLM (*QPglm-dsr09-3*), GLAB (*QGlab-dsr09-3*) and GLAM (*QGlam-dsr09-3*). Sabadin *et al.,* [83] also reported co-location of QTL for grain yield and stay-green at the *QGY-dsr09-3*. Similarly, *QGY-dsr04-1* QTL on SBI-04 was co-located with QTL for GLB (*QGlb-dsr04-1*) and GLM (*QGlm-dsr04-1*). A positive impact of stay-green on grain yield under terminal drought was reported [7, 8, 50, 74]. Co-location between an apparently novel stay-green QTL and grain yield QTL in SBI-04 and SBI-09 suggests there is potential for indirect selection for improved grain yield based on stay-green under post-flowering drought stress.

### Association of genic-SSRs with stay green QTL

Association of genic markers with quantitative trait loci increase our understanding of genes influencing desired traits [75, 85–87]. In the present study, 14 genic markers were found either as QTL locus or closely linked with the QTL of ten traits studied, thus providing simple PCR based markers for MAS of these traits (Table 3; Figure 1). A genic marker *XnhsbSFCILP67* (Sb03g028240) encoding Indole-3-acetic acid-amido synthetase GH3.5 (Additional file 5: Table S3), was co-located with QTL for GLB, GLM, PGLM and GLAM on SBI-03. The expression of this gene was induced by auxin [88]. An EST derived marker *Stgnhsbm19*, derived from a Chlorophyll A-B binding protein gene (CAB gene) was co-located with QTL for GLM and PGLM. CAB proteins are essential pigment binding proteins of light harvesting complex (LHC) which is involved in photosynthesis [89, 90]. CAB proteins also play an important role in plant development [91] and leaf senescence [73]. Therefore, the CAB gene may be a candidate for traits, which probably are influenced by photosynthesis. However, further detailed genetic analysis of CAB gene should reveal its molecular mechanisms underlying stay-green and other related traits. Similarly, a genic marker *S18* (Sb09g030740) on SBI-09 was closely linked with *QSpadb-dsr09-3*, *QGlb-dsr09-3*, *QGlm-dsr09-3*, *QGlab-dsr09-3*, *QGlam-dsr09-3* and *QPglm-dsr09-3* QTL controlling SPADB, GLB, GLM, GLAB, GLAM, PGLM and GY. This gene encodes Kelch-related proteins, which are involved in cell development and programmed cell death. Biological studies indicate role of these proteins in phyto-hormone response, embryo development and programmed cell death [92, 93].

### Co-location of stay-green QTL and genes involved in the chlorophyll metabolism

**Figure 2:**
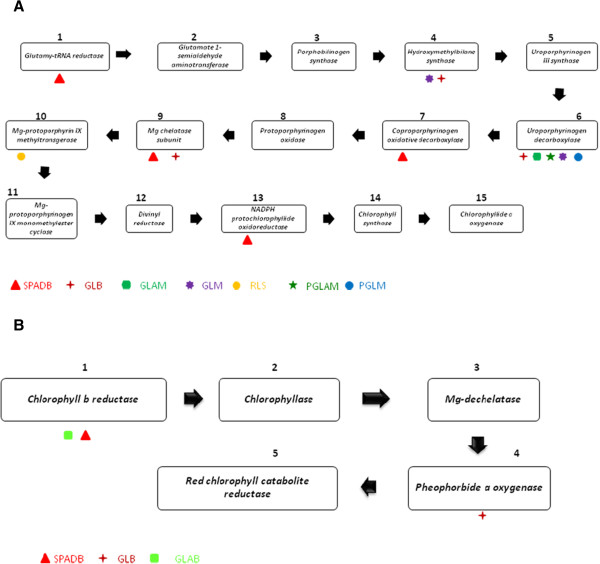
**Co-location of QTL for stay-green and genes controlling chlorophyll metabolism.** *SPADB*: SPAD values at Booting, *SPADM*: SPAD values at maturity, *GLB*: Green leaves at booting, *GLM*: Green leaves at maturity, *PGLM*: Percent green leaves retained at maturity, *GLAB*: Green leaf area at booting, *GLAM*: Green leaf area at maturity, *PGLAM*: Per cent green leaf area at maturity, *RLS*: Rate of leaf senescence, *GY*: Grain yield per panicle. **A**. Enzymes involved in Chlorophyll Biosynthesis. **B**. Enzymes involved in Chlorophyll degradation.

Comparative genomic analysis provides an excellent opportunity to learn from model crop plants like Arabidopsis to deduce information on the relevant genes involved in a trait expression. In chlorophyll metabolism, 15 enzymes catalyzing chlorophyll biosynthesis and five enzymes catalyzing its degradation (Figure 2.) have been identified and the genes coding these enzymes have been cloned from this model plant. Search was made in the genomic region of the sorghum stay-green QTL for the existence of genes involved in chlorophyll metabolism identified in model plant. Many of the stay-green QTL of the present study overlapped with the genes controlling enzymes involved in chlorophyll metabolism (Additional file 6: Table S2). For instance, the QTL *QGLM-dsr04-1*, *QGLB-dsr04-1*, *QGLB-dsr03*, *QGLAM-dsr03*, *QPGLAM-dsr03*, *QGLM-dsr03*, *QPGLM-dsr03*, *QGLB-dsr03a*, *QGLM-dsr03*, *QGLM-dsr03*, *QGLM-dsr03*, *QGLAB-dsr03a*, *QGLB-dsr03a* and *QRLS-dsr10-1* co-located with the genes involved in chlorophyll biosynthesis, while the QTL *QSPADB-dsr06-1*, *QGLB-dsr04-3*, *SPADB-dsr01-1*, *QGLM-dsr02-3a*, *QSPADB-dsr05*, *QGLB-dsr03b*, *QSPADB-dsr03* and *QGLB-dsr03-1a* found near to genes involved in chlorophyll biosynthesis and degradation. Comparing stay-green QTL and the genes controlling chlorophyll biosynthesis and degradation in rice, the genetic basis for stay-green could be through up-regulation of chlorophyll biosynthesis- genes and down-regulation of chlorophyll degradation genes [94]. Similar mechanism as proposed in rice may also operate in sorghum in the expression of stay-green traits.

## Conclusions

We validated important stay-green QTL reported earlier and detected new QTL influencing stay-green. Major QTL identified are reliable and can be employed for MAS to improve drought tolerance of post-rainy sorghum varieties. *Stg2*, *Stg3* and *StgB* are key QTL identified in the study for marker-assisted selection to improve terminal drought tolerance. The QTL linked markers would help sorghum breeders to accumulate desirable allelic combinations and accelerate development of improved drought tolerant sorghum varieties. Understanding the genetic basis and molecular mechanisms of stay-green and cloning of the genes responsible will have great impact in improving crop productivity under drought stress not only in sorghum, but in other major cereal crops as well.

## Electronic supplementary material

Additional file 1: Table S4: Monthly maximum and minimum temperatures, rainfall (mm and number of rainy days), sunshine hours, wind speed and pan evaporation recorded at DSR during the 2006–2009 experiment period. (DOCX 15 KB)

Additional file 2: Figure S1: Projection of 91 QTL identified for the 11 traits in M35-1 x B35 RIL mapping population on the physical map of Mace and Jordan (2011). (DOC 190 KB)

Additional file 3: Figure S2: Frequency distribution of 245 RILs for 10 traits (mean of three seasons). (PPT 662 KB)

Additional file 4: Table S1: The F value of ANOVA for genotype, environment and genotype x environment interaction for 10 traits studied in the RIL population. (DOCX 13 KB)

Additional file 5: Table S3: Putative function of genic-SSR markers mapped in the present study. (DOC 144 KB)

Additional file 6: Table S2: Co-location of stay-green QTLs and genes controlling chlorophyll metabolism. (DOC 54 KB)
