# Supplementary material for: Detection and validation of stay-green QTL in post-rainy sorghum involving widely adapted cultivar, M35-1 and a popular stay-green genotype B35
Source: BMC Genomics. 2014 Oct 18;15(1):909. doi: 10.1186/1471-2164-15-909 (PMC4219115; doi:10.1186/1471-2164-15-909)
Supplement: Supplementary file 1 — Additional file 1: Table S4: Monthly maximum and minimum temperatures, rainfall (mm and number of rainy days), sunshine hours, wind speed and pan evaporation recorded at DSR during the 2006–2009 experiment period. (DOCX 15 KB) [file 12864_2014_6617_MOESM1_ESM.docx]

**Supplementary Table 4. Monthly maximum and minimum temperatures, rainfall (mm and number of rainy days), sunshine hours, wind speed and pan evaporation recorded at DSR during the 2006-2009 experiment period**

| **MONTHLY METEOROLOGICAL DATA RECORDED AT DSR, RAJENDRANAGAR DURING 2006-2007** | | | | | | | |
| --- | --- | --- | --- | --- | --- | --- | --- |
| **Month** | **Temperature (^o^C)** | | **Rain fall (mm)** | **Rainy days** | **Sun shine (hrs)** | **Wind speed (km/hr)** | **Evaporation (mm)** |
|  | MAX | MIN |  |  |  |  |  |
| OCT | 30.8 | 20.6 | 17.5 | 2 | 7 | 4.0 | 4.4 |
| NOV | 28.7 | 18.6 | 27.3 | 2 | 6 | 3.2 | 4.1 |
| DEC | 28.6 | 13.3 | 0.0 | 0 | 9 | 3.2 | 4.4 |
| JAN | 29.4 | 13.8 | 0.0 | 0 | 9 | 3.3 | 4.3 |
| FEB | 31.4 | 15.5 | 0.0 | 0 | 9 | 4.6 | 4.9 |
| MAR | 35.7 | 20.5 | 0.0 | 0 | 8 | 4.9 | 6.6 |
| **Mean/Total** | **30.8** | **17.1** | **44.8** | **4.0** | **48.1** | **3.9** | **28.8** |
| **MONTHLY METEOROLOGICAL DATA RECORDED AT DSR, RAJENDRANAGAR DURING 2007-2008** | | | | | | | |
| **Month** | **Temperature (^o^C)** | | **Rain fall (mm)** | **Rainy days** | **Sun shine (hrs)** | **Wind speed (km/hr)** | **Evaporation (mm)** |
|  | MAX | MIN |  |  |  |  |  |
| OCT | 30.6 | 19.4 | 14.8 | 3.0 | 6.2 | 3.0 | 5.6 |
| NOV | 29.5 | 13.8 | 15.8 | 1.0 | 8.4 | 3.0 | 5.0 |
| DEC | 29.6 | 14.3 | 0.0 | 0.0 | 7.9 | 3.2 | 4.6 |
| JAN | 30.3 | 13.0 | 0.0 | 0.0 | 9.2 | 3.5 | 4.8 |
| FEB | 30.5 | 18.1 | 74.4 | 3.0 | 7.5 | 4.0 | 4.6 |
| MAR | 33.4 | 19.2 | 98.0 | 4.0 | 7.3 | 4.1 | 5.8 |
| **Mean/Total** | **30.6** | **16.3** | **203.0** | **11.0** | **46.6** | **3.5** | **30.4** |
| **MONTHLY METEOROLOGICAL DATA RECORDED AT DSR, RAJENDRANAGAR DURING 2008-2009** | | | | | | | |
| **Month** | **Temperature (^o^C)** | | **Rain fall (mm)** | **Rainy days** | **Sun shine (hrs)** | **Wind speed (km/hr)** | **Evaporation (mm)** |
|  | MAX | MIN |  |  |  |  |  |
| OCT | 31.4 | 19.9 | 53.6 | 2 | 7 | 3.1 | 5.9 |
| NOV | 29.6 | 16.6 | 12.6 | 2 | 7 | 3.6 | 5.9 |
| DEC | 29.5 | 14.3 | 0.0 | 0 | 8 | 2.4 | 5.3 |
| JAN | 30.0 | 12.0 | 0.0 | 0 | 9 | 3.3 | 4.3 |
| FEB | 32.0 | 16.0 | 0.0 | 0 | 9 | 4.6 | 4.9 |
| MAR | 36.0 | 25.0 | 0.0 | 0 | 8 | 4.9 | 6.6 |
| **Mean/Total** | **31.4** | **17.3** | **66.2** | **4** | **49** | **3.7** | **32.9** |
| ***Average*** | ***30.9*** | ***16.9*** | ***104.7*** | ***6.3*** | ***47.9*** | ***3.7*** | ***30.7*** |
