# Supplementary material for: Detection and validation of stay-green QTL in post-rainy sorghum involving widely adapted cultivar, M35-1 and a popular stay-green genotype B35
Source: BMC Genomics. 2014 Oct 18;15(1):909. doi: 10.1186/1471-2164-15-909 (PMC4219115; doi:10.1186/1471-2164-15-909)
Supplement: Supplementary file 2 — Additional file 2: Figure S1: Projection of 91 QTL identified for the 11 traits in M35-1 x B35 RIL mapping population on the physical map of Mace and Jordan (2011). (DOC 190 KB) [file 12864_2014_6617_MOESM2_ESM.doc]

Supplementary Figure 1. Projection of 91 QTL identified for the 11 traits in M35-1 x B35 RIL mapping population on the physical map of Mace and Jordan (2011). The length of the vertical bars indicates physical intervals of each QTL based the positions of flanking markers. Scale in Million bases (Mb)

Supplementary Figure 1. Projection of 91 QTL identified for the 11 traits in M35-1 x B35 RIL mapping population on the physical map of Mace and Jordan (2011). The length of the vertical bars indicates physical intervals of each QTL based the positions of flanking markers. Scale in Million bases (Mb)
