# Supplementary material for: Detection and validation of stay-green QTL in post-rainy sorghum involving widely adapted cultivar, M35-1 and a popular stay-green genotype B35
Source: BMC Genomics. 2014 Oct 18;15(1):909. doi: 10.1186/1471-2164-15-909 (PMC4219115; doi:10.1186/1471-2164-15-909)
Supplement: Supplementary file 3 — Additional file 3: Figure S2: Frequency distribution of 245 RILs for 10 traits (mean of three seasons). (PPT 662 KB) [file 12864_2014_6617_MOESM3_ESM.ppt]

## Slide 1
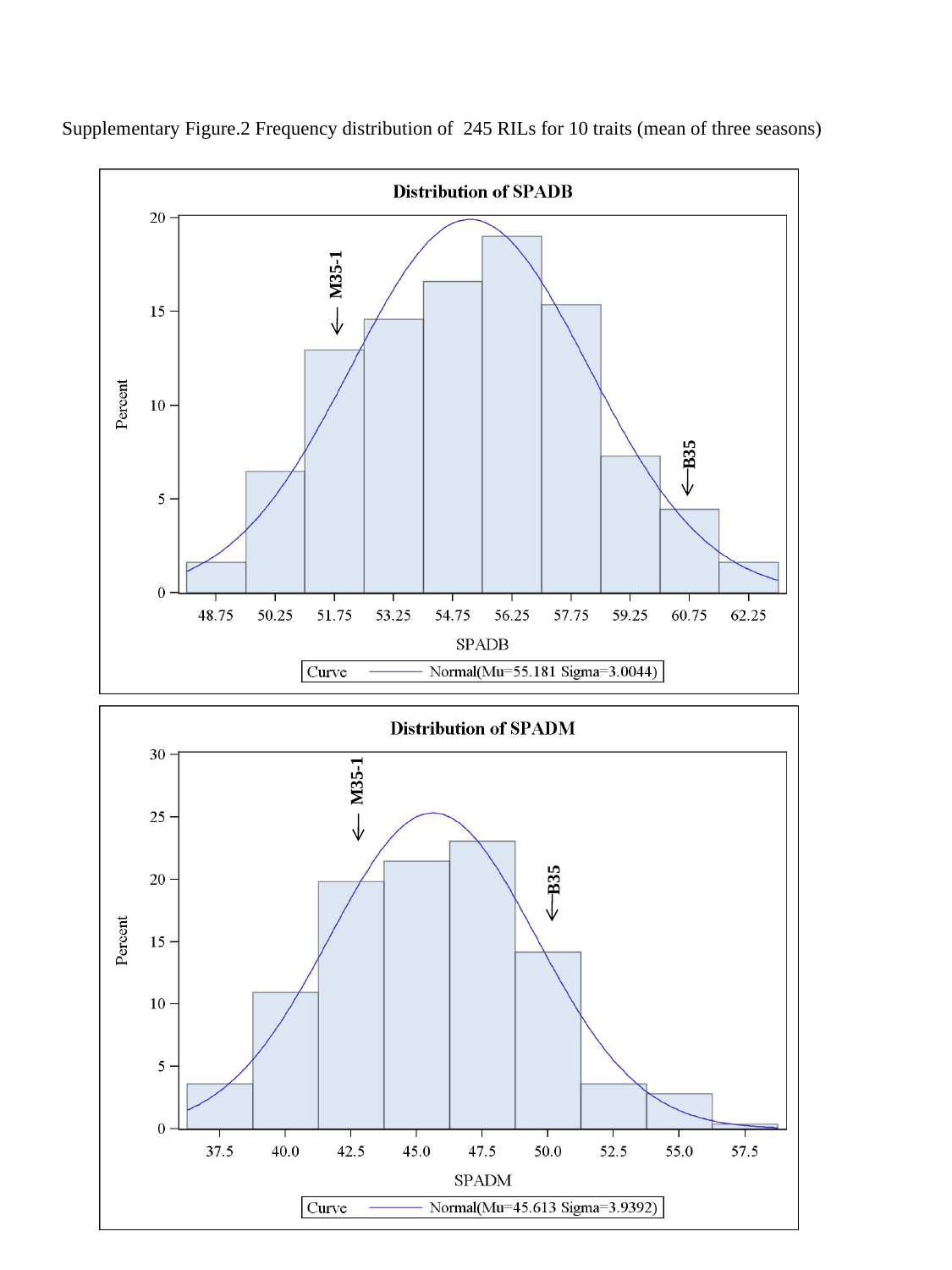

Supplementary Figure.2 Frequency distribution of 245 RILs for 10 traits (mean of three seasons)
M35-1
B35
M35-1
B35

## Slide 2
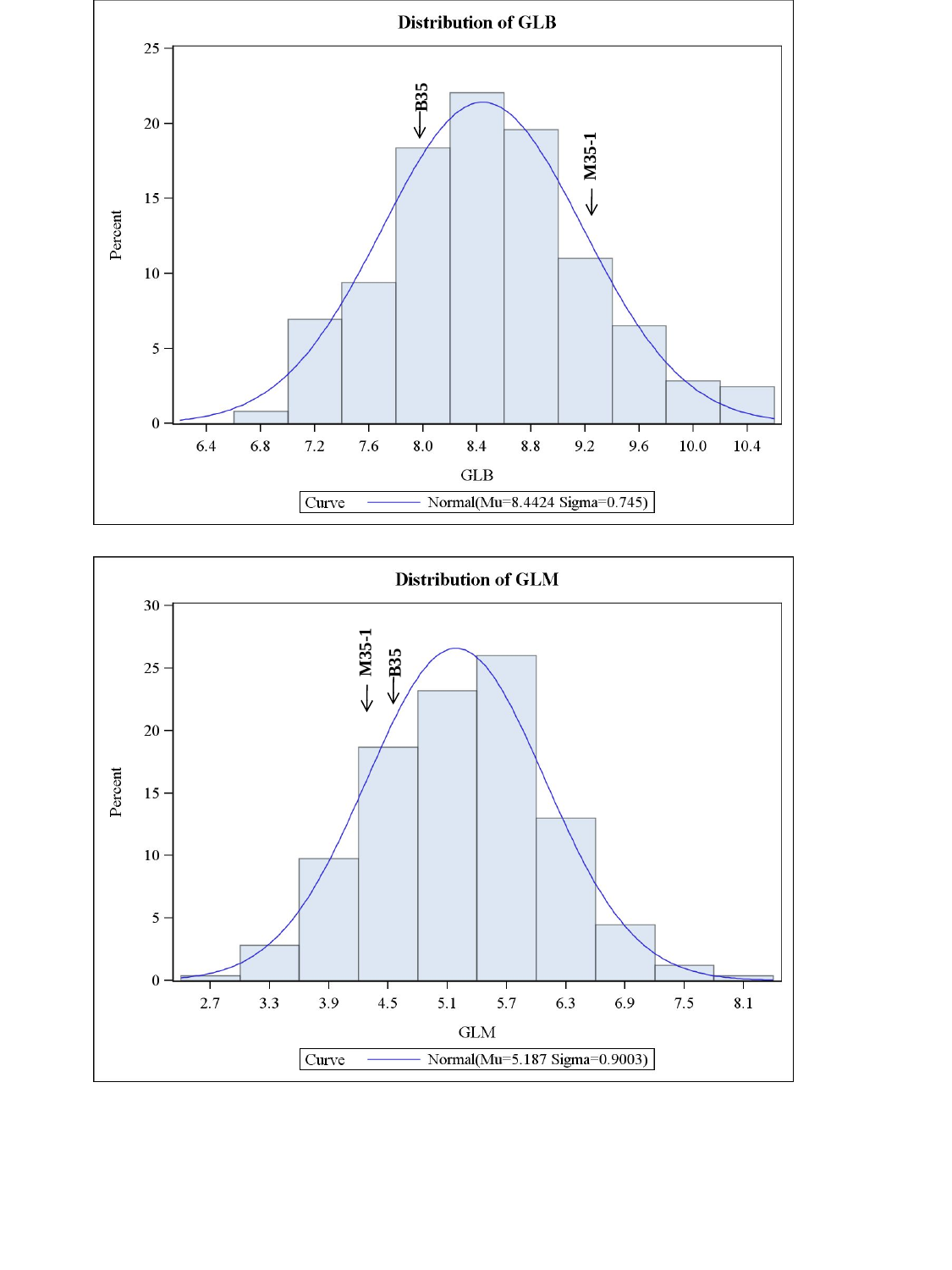

B35
M35-1
M35-1
B35

## Slide 3
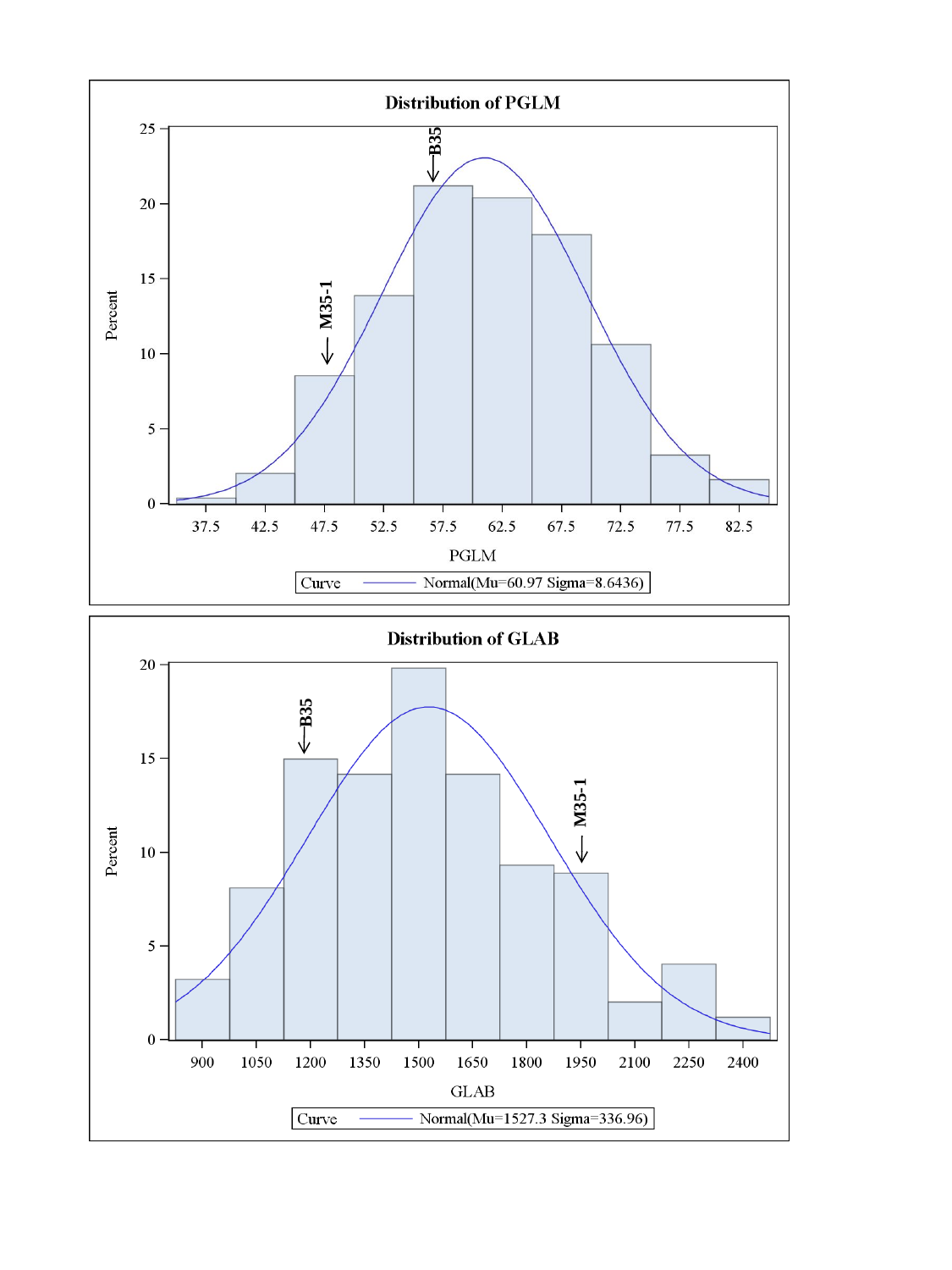

B35
M35-1
B35
M35-1

## Slide 4
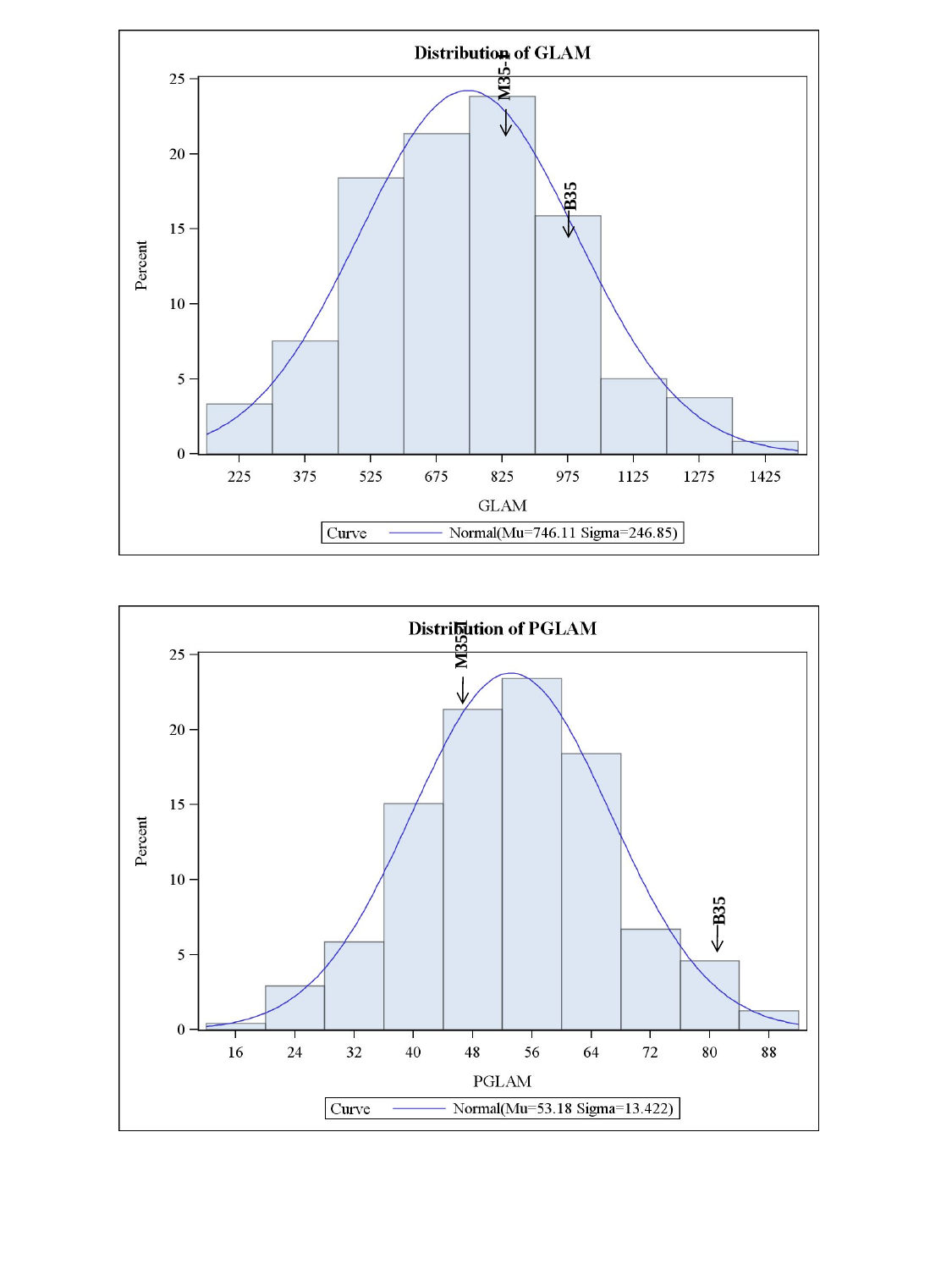

M35-1
B35
M35-1
B35

## Slide 5
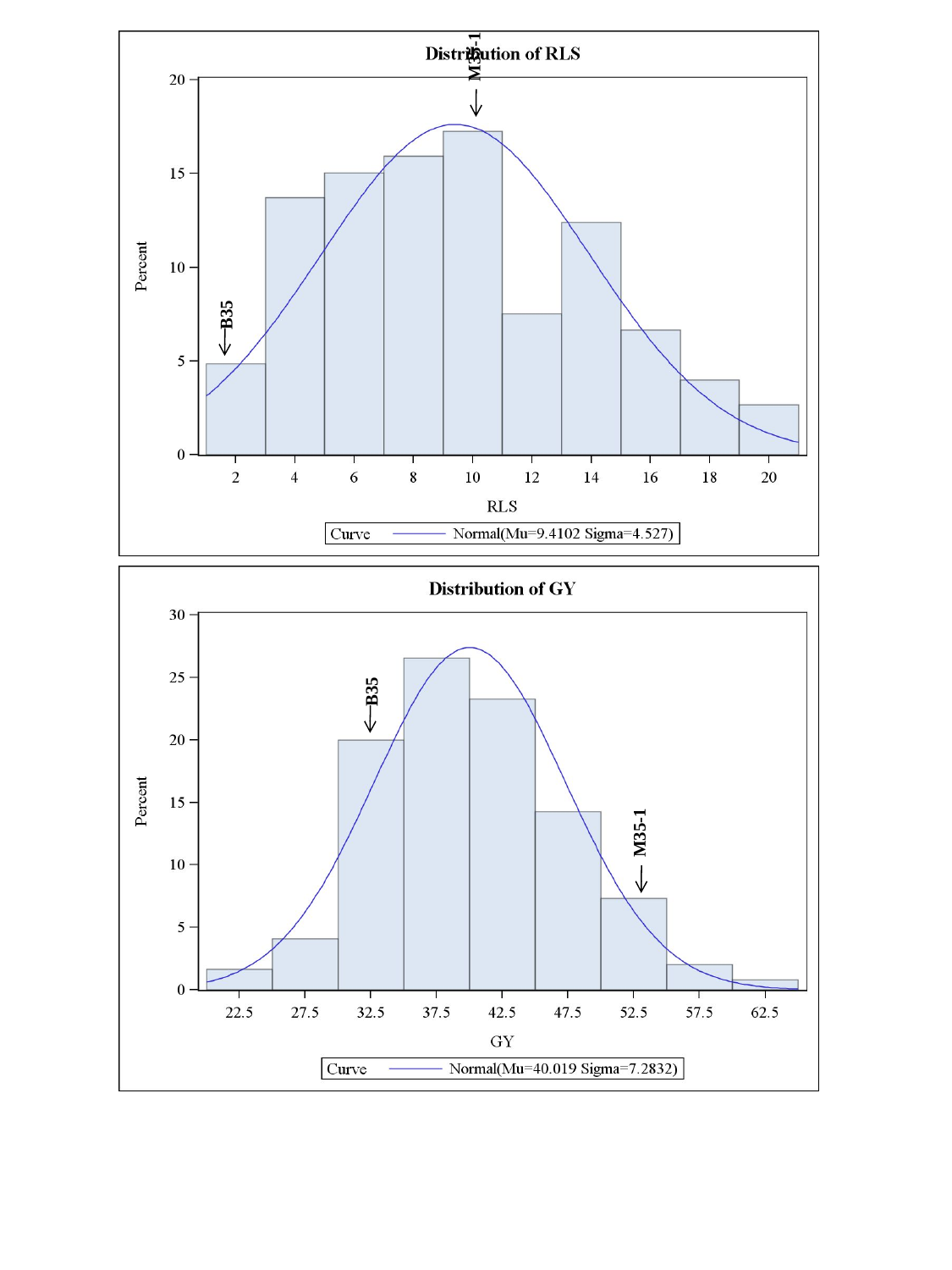

M35-1
B35
B35
M35-1
