# Supplementary material for: Detection and validation of stay-green QTL in post-rainy sorghum involving widely adapted cultivar, M35-1 and a popular stay-green genotype B35
Source: BMC Genomics. 2014 Oct 18;15(1):909. doi: 10.1186/1471-2164-15-909 (PMC4219115; doi:10.1186/1471-2164-15-909)
Supplement: Supplementary file 4 — Additional file 4: Table S1: The F value of ANOVA for genotype, environment and genotype x environment interaction for 10 traits studied in the RIL population. (DOCX 13 KB) [file 12864_2014_6617_MOESM4_ESM.docx]

**Supplementary Table 1. The F value of ANOVA for genotype, environment and genotype x environment interaction for 10 traits studied in the RIL population**

|  | **Genotype** | | | **Environment** | | | **Genotype x environment** | | |
| --- | --- | --- | --- | --- | --- | --- | --- | --- | --- |
| **Trait** | ***df*** | ***F*-value** | ***P*-value** | ***df*** | ***F*-value** | ***P*-value** | ***df*** | ***F*-value** | ***P*-value** |
| **SPADB** | 246 | 3.72 | 0.00 | 2 | 63.12 | 0.00 | 237 | 1.72 | 0.00 |
| **SPADM** | 246 | 2.47 | 0.00 | 2 | 555.98 | 0.00 | 237 | 1.83 | 0.00 |
| **GLB** | 246 | 5.38 | 0.00 | 2 | 326.64 | 0.00 | 237 | 1.74 | 0.00 |
| **GLM** | 246 | 3.74 | 0.00 | 2 | 544.92 | 0.00 | 237 | 1.62 | 0.00 |
| **PGLM** | 246 | 2.79 | 0.00 | 2 | 463.30 | 0.00 | 237 | 1.68 | 0.00 |
| **GLAB** | 246 | 7.40 | 0.00 | 2 | 112.58 | 0.00 | 237 | 1.74 | 0.00 |
| **GLAM** | 246 | 2.57 | 0.00 | 2 | 197.40 | 0.00 | 237 | 1.60 | 0.00 |
| **PGLAM** | 246 | 1.69 | 0.00 | 2 | 319.73 | 0.00 | 237 | 1.25 | 0.04 |
| **RLS** | 246 | 2.38 | 0.00 | 2 | 350.00 | 0.00 | 237 | 1.05 | 0.00 |
| **GY** | 246 | 2.99 | 0.00 | 2 | 62.20 | 0.00 | 237 | 1.35 | 0.00 |
