# Supplementary material for: Detection and validation of stay-green QTL in post-rainy sorghum involving widely adapted cultivar, M35-1 and a popular stay-green genotype B35
Source: BMC Genomics. 2014 Oct 18;15(1):909. doi: 10.1186/1471-2164-15-909 (PMC4219115; doi:10.1186/1471-2164-15-909)
Supplement: Supplementary file 5 — Additional file 5: Table S3: Putative function of genic-SSR markers mapped in the present study. (DOC 144 KB) [file 12864_2014_6617_MOESM5_ESM.doc]

**Supplementary Table 3. Putative function of genic-SSR markers mapped in the present study**

| **S. No.** | **Locus** | **EST Acc. No./ Unigene ID** | **LG** | **Sorghum gene** | **Location** | **Putative function** |
| --- | --- | --- | --- | --- | --- | --- |
| 1 | Undhsbm1 | Sbi.63 | SBI-01 | Sb01g001180 | chromosome_1: 1058885 - 1061083 | similar to Cytochrome P450 71E1 |
| 2 | Undhsbm68 | Sbi.2622 | SBI-01 | Sb01g008290 | chromosome_1:7157207 - 7158750 | similar to Putative uncharacterized protein OJ1124_H03.7 |
| 3 | Undhsbm90 | Sbi.3399 | SBI-01 | Sb01g011750 | chromosome_1:10629875 - 10633877 | similar to Glutamine amidotransferase class-I family protein |
| 4 | S23 | CNL182 | SBI-01 | Sb01g012740 | chromosome_1:11730337 - 11733690 | similar to Tubulin beta-2/beta-3 chain |
| 5 | Xisep1038 | AW565964 | SBI-01 | Sb01g018800 | chromosome_1:19721291 - 19725402 | similar to Zinc finger, C3HC4 type family protein |
| 6 | Xisep1028 | AW747772 | SBI-01 | Sb01g029930 | chromosome_1:52056562 - 52057686 | similar to Putative uncharacterized protein |
| 7 | Undhsbm24 | Sbi.736 | SBI-01 | Sb01g036740 | chromosome_1:60346941 - 60350979 | similar to Ethylene-insensitive-3-like protein |
| 8 | Undhsbm195 | Sbi.8865 | SBI-01 | Sb01g040640 | chromosome_1:63956959 - 63957486 | similar to Os08g0239000 protein |
| 9 | Undhsbm82 | Sbi.3125 | SBI-02 | Sb02g000230 | chromosome_2:129263 - 131530 | similar to Tic62 protein, putative, expressed |
| 10 | Undhsbm154 | Sbi.7032 | SBI-02 | Sb02g014690 | chromosome_2: 28678566 - 28691538 | similar to Flowering time control protein FCA gamma-like |
| 11 | Undhsbm159 | Sbi.7366 | SBI-02 | Sb02g026360 | chromosome_2: 61465916 - 61469106 | similar to Putative Avr9 elicitor response protein |
| 12 | Xisep841 | BE358373 | SBI-02 | Not in gene | chromosome_2:70840382-70940481 | -- |
| 13 | Drenhsbm32 | CF770135 | SBI-02 | Sb02g026360 | chromosome_2:61465916 - 61469106 | similar to Putative Avr9 elicitor response protein |
| 14 | Xisep522 | BE361646 | SBI-02 | Sb02g043950 | chromosome_2: 77749505 - 77754014 | similar to Os07g0693600 protein |
| 15 | Xisep101 | AW679887 | SBI-03 | Sb03g006870 | chromosome_3:7097311 - 7098093 | similar to Heat shock protein 17.2 |
| 16 | Xisep1012 | BE360971 | SBI-03 | Sb03g007915 | chromosome_3:8300368 - 8301801 | similar to Putative uncharacterized protein |
| 17 | Xisep114 | AW745467 | SBI-03 | Sb03g025455 | chromosome_3:51176407 - 51176664 | similar to Cysteine-rich extensin-like protein-2 |
| 18 | Undhsbm56 | Sbi.2161 | SBI-03 | Sb03g001650 | chromosome_3:1479755 - 1482190 | similar to Putative esterase |
| 19 | Undhsbm52 | Sbi.2012 | SBI-03 | Not in gene | chromosome_2:61933666 - 62033865 | -- |
| 20 | Stgnhsbm2 | CX616697 | SBI-03 | Sb03g032260 | chromosome_3:60693795 - 60695579 | myb-related protein Hv33, putative, expressed |
| 21 | Stgnhsbm9 | CD223691 | SBI-03 | Sb03g033550 | chromosome_3:61820363 - 61822645 | similar to Putative uncharacterized protein |
| 22 | Drenhsbm10 | CF772123 | SBI-03 | Sb03g046730 | chromosome_3: 73788493 - 73790878 | similar to Probable ubiquitin-fold modifier 1 precursor |
| 23 | Undhsbm164 | Sbi.7738 | SBI-03 | Not in gene | chromosome_3:6589749 - 6589768 | -- |
| 24 | Undhsbm314 | Sbi10880 | SBI-03 | Sb03g040910 | chromosome_3: 68417828 - 68418925 | similar to Putative uncharacterized protein |
| 25 | Undhsbm209 | Sbi.9424 | SBI-03 | Sb03g041550 | chromosome_3: 69056445 - 69059782 | similar to Putative uncharacterized protein |
| 26 | Xisep224 | AW563693 | SBI-04 | Sb04g000840 | chromosome_4: 698389 - 701098 | similar to Putative uncharacterized protein |
| 27 | Xisep948 | BE358472 | SBI-04 | Sb04g001130 | chromosome_4: 966713 - 968581 | similar to Catalase isozyme 3 |
| 28 | Xisep203 | BE359080 | SBI-04 | Sb04g008600 | chromosome_4: 9998074 - 10011232 | simillar to class III peroxidase |
| 29 | Undhsbm66 | Sbi.2553 | SBI-04 | Sb04g000540 | chromosome_4: 372053 - 375378 | similar to Putative uncharacterized protein OJA1212_C06.19 |
| 30 | Undhsbm210 | Sbi.9457 | SBI-04 | Sb04g025890 | chromosome_4: 55666981 - 55674595 | similar to Leucine-rich repeat-like protein |
| 31 | Xisep524 | BE358788 | SBI-04 | Sb04g037640 | chromosome_4: 67229617 - 67232557 | similar to Putative fibrillarin protein |
| 32 | Xisep234 | AW680664 | SBI-04 | Sb04g036660 | chromosome_4: 66478839 - 66483569 | similar to Putative GTPase activating protein |
| 33 | Undhsbm265 | Sbi.11534 | SBI-04 | Sb04g037260 | chromosome_4: 66934638 - 66936987 | similar to Auxin-responsive protein IAA10 |
| 34 | Xisep1208 | AW287725 | SBI-05 | Sb05g000940 | chromosome_5: 879883 - 881239 | Hypoxia induced protein conserved region containing protein, expressed |
| 35 | Stgnhsbm46 | CX611411 | SBI-05 | Sb05g009350 | chromosome_5:18472314 - 18475420 | similar to Alcohol dehydrogenase 2 |
| 36 | Xisep1107 | BE125211 | SBI-05 | Sb05g006520 | chromosome_5: 10622824 - 10628985 | similar to Expressed protein |
| 37 | Xisep443 | BE355584 | SBI-06 | Sb06g026670 | chromosome_6: 55680173 - 55680640 | similar to H0307D04.12 protein |
| 38 | Xisep427 | AW563837 | SBI-06 | Sb06g031060 | chromosome_6: 59441882 - 59445806 | similar to OSJNBa0060D06.16 protein |
| 39 | Xisep131 | BE357921 | SBI-07 | Sb07g000380 | chromosome_7: 170550 - 171704 | similar to Farnesylated protein 3 |
| 40 | Xisep805 | BE352923 | SBI-07 | Sb07g001680 | chromosome_7: 1933118 - 1934060 | similar to Putative uncharacterized protein |
| 41 | Xisep328 | BE359052 | SBI-07 | Sb07g004290 | chromosome_7: 5440507 - 5442963 | 40S ribosomal protein S3 |
| 42 | Drenhsbm16 | CF771342 | SBI-08 | Sb08g000945 | chromosome_8: 873750 - 880414 | similar to Putative uncharacterized protein OSJNBa0041F13.17 |
| 43 | Undhsbm109 | Sbi.4081 | SBI-08 | Sb08g020760 | chromosome_8: 52053371 - 52059284 | similar to Tetratricopeptide repeat protein, putative, expressed |
| 44 | Xisep550 | AW746901 | SBI-09 | Sb09g004270 | chromosome_9: 5060151 - 5063515 | spliceosomal protein snRNP-U1A/U2B |
| 45 | Undhsbm178 | Sbi.8301 | SBI-09 | Sb09g028300 | chromosome_9: 57219361 - 57222245 | Serine/threonine protein kinase |
| 46 | Stgnhsbm19 | CN144249 | SBI-09 | Sb09g028720 | chromosome_9: 57560635 - 57561977 | similar to Chlorophyll A-B binding protein (CAB), putative |
| 47 | S18 | CNL177 | SBI-09 | Sb09g030740 | chromosome_9: 59373345 - 59375990 | similar to Putative uncharacterized protein |
| 48 | Undhsbm217 | Sbi.9792 | SBI-10 | Sb10g000390 | chromosome_10: 157912 - 163241 | similar to Putative uncharacterized protein |
| 49 | Xisep604 | AW563246 | SBI-10 | Sb10g000230 | chromosome_10: 31694 - 32694 | similar to Plastocyanin, chloroplast precursor |
| 50 | Undhsbm105 | Sbi.4002 | SBI-10 | Sb10g000470 | chromosome_10: 274080 - 278793 | similar to Putative uncharacterized protein |
| 51 | Xisep625 | AW677135 | SBI-10 | Sb10g021750 | chromosome_10: 48098018 - 48101431 | similar to Putative uncharacterized protein |
| 52 | Xdhsbm1025 | Sbi17757 | SBI-10 | Sb10g026120 | chromosome_10: 55468789 - 55472702 | similar to Integral membrane protein-like |
| 53 | Xisep630 | AW922806 | SBI-10 | Sb10g027610 | chromosome_10: 57400262 - 57401004 | similar to EF-hand Ca2+-binding protein CCD1 |
| 54 | Xisep1011 | AW283079 | SBI-10 | Sb10g031090 | chromosome_10: 60742768 - 60746789 | simillar to cell elongation protein diminuto-related |
