# Supplementary material for: Detection and validation of stay-green QTL in post-rainy sorghum involving widely adapted cultivar, M35-1 and a popular stay-green genotype B35
Source: BMC Genomics. 2014 Oct 18;15(1):909. doi: 10.1186/1471-2164-15-909 (PMC4219115; doi:10.1186/1471-2164-15-909)
Supplement: Supplementary file 6 — Additional file 6: Table S2: Co-location of stay-green QTLs and genes controlling chlorophyll metabolism. (DOC 54 KB) [file 12864_2014_6617_MOESM6_ESM.doc]

**Supplementary Table 2 : Co-location of staygreen QTLs and genes controlling chlorophyll metabolism**

| **Chromosome** | **QTL** | **Sorghum gene ID** | **Location** | **Arabidopsis gene** | **Description** | **Functional role** | **Reference** |
| --- | --- | --- | --- | --- | --- | --- | --- |
| SBI-06 | QSPADB-dsr06-1 (Xcup12-Xcup37) | Sb06g030160 | 6: 58542371-58545711 | [AT1G09940](http://www.arabidopsis.org/servlets/TairObject?type=locus&name=AT1G09940) (HEMA2) | Glutamyl-tRNA reductase | Chlorophyll biosynthesis | Beale (2005) |
|  |  | Sb06g032740 | 6: 60839090-60844506 | AT5G13630 (CHLH) | Mg chelatase H subunit | Chlorophyll biosynthesis | Beale (2005) |
|  |  | Sb06g033030 | 6: 61062710-61064337 | AT5G54190 (PORA) | NADPH:protochlorophyllide oxidoreductase | Chlorophyll biosynthesis | Beale (2005) |
|  |  | Sb06g028140 | 6: 56988364-56992323 | AT1G03475 (HEMF1) | Coproporphyrinogen oxidative decarboxylase | Chlorophyll biosynthesis | Beale (2005) |
|  |  | Sb06g028140 | 6:56988364-56992323 | AT1G03475 | coproporphyrinogen oxidase | Chlorophyll biosynthesis | Ishikawa et al. (2001) |
|  |  | Sb06g028750 | 6:57494696-57495877 | AT2G20180 | PHYTOCHROME INTERACTING FACTOR 3-LIKE 5 | Chlorophyll biosynthesis | Moon et al. (2008) |
|  |  | Sb06g028880 | 6:57610715-57622704 | AT2G34660 | ARABIDOPSIS THALIANA MULTIDRUG RESISTANCE-ASSOCIATED PROTEIN 2 | Chlorophyll degradation | Frelet-Barrand et. al. (2008) |
|  |  | Sb06g030230 | 6:58663766-58664958 | AT2G21970 | STRESS ENHANCED PROTEIN 2 | Chlorophyll biosynthesis | Heddad et al. (2000) |
|  |  | Sb06g032690 | 6:60809627-60810528 | AT1G08520 (CHLD) | Magnesium chelatase | Chlorophyll biosynthesis | Kobayashi et al. (2008) |
| SBI-04 | QGLM-dsr04-1;  QGLB-dsr04-1  (Xisp230-gpsb050) | Sb04g004630 | 4:4443739-4447383 | AT5G08280 (HEMC) | Hydroxymethylbilane synthase | Chlorophyll biosynthesis | Beale (2005) |
|  |  | Sb04g004640 | 4:4447744-4450352 | AT5G08280 (HEMC) | Hydroxymethylbilane synthase | Chlorophyll biosynthesis | Beale (2005) |
| SBI-03 | QGLB-dsr03; QGLAM-dsr03; QPGLAM-dsr03;  QGLM-dsr03; QPGLM-dsr03; QGLB-dsr03a  QGLM-dsr03; QGLM-dsr03; QGLM-dsr03; QGLAB-dsr03a  QGLB-dsr03a | Sb03g028330 | 3:56112540-56117698 | AT3G14930 (HEME1) | Uroporphyrinogen decarboxylase | Chlorophyll biosynthesis | Beale (2005) |
|  | QGLAB-dsr03b  QSPADB-dsr03 | Sb03g037870 | 3: 65785702-65789235 | AT5G17770 (ATCBR) | Chlorophyll b reductase | Chlorophyll degradation | Hortensteiner (2006) |
| SBI-01 | QGLB-dsr01-1a | Sb01g003860 | 1: 3114774-3124068 | AT1G08520 (CHLD) | Mg chelatase D subunit | Chlorophyll biosynthesis | Beale (2005) |
|  |  | Sb01g003860 | 1: 3114774-3124068 | AT4G18480 (CHLI1) | Mg chelatase I subunit | Chlorophyll biosynthesis | Beale (2005) |
|  |  | Sb01g004440 | 1:3550546-3553033 | AT4G25650(ACD1-LIKE) | Pheophorbide a oxygenase | Chlorophyll degradation | Hortensteiner (2006) |
|  |  | Sb01g004450 | 1:3557127-3559349 | AT4G25650(ACD1-LIKE) | Pheophorbide a oxygenase | Chlorophyll degradation | Hortensteiner (2006) |
| SBI-10 | QRLS-dsr10-1 | Sb10g002100 | 10:1769123-1770560 | AT4G25080 (CHLM) | Mg-protoporphyrin IX methyltransferase | Chlorophyll biosynthesis | Beale (2005) |
